# Microglia: Key Players in Retinal Ageing and Neurodegeneration

**DOI:** 10.3389/fncel.2022.804782

**Published:** 2022-03-17

**Authors:** Li Guo, Soyoung Choi, Priyanka Bikkannavar, M. Francesca Cordeiro

**Affiliations:** ^1^Institute of Ophthalmology, University College London, London, United Kingdom; ^2^Imperial College Ophthalmology Research Group, Imperial College London, London, United Kingdom

**Keywords:** microglia, morphology, phenotypes, phagocytosis, retina, ageing, retinal neurodegenerative disease

## Abstract

Microglia are the resident immune cells of the central nervous system (CNS) and play a key role in maintaining the normal function of the retina and brain. During early development, microglia migrate into the retina, transform into a highly ramified phenotype, and scan their environment constantly. Microglia can be activated by any homeostatic disturbance that may endanger neurons and threaten tissue integrity. Once activated, the young microglia exhibit a high diversity in their phenotypes as well as their functions, which relate to either beneficial or harmful consequences. Microglial activation is associated with the release of cytokines, chemokines, and growth factors that can determine pathological outcomes. As the professional phagocytes in the retina, microglia are responsible for the clearance of pathogens, dead cells, and protein aggregates. However, their phenotypic diversity and phagocytic capacity is compromised with ageing. This may result in the accumulation of protein aggregates and myelin debris leading to retinal neuroinflammation and neurodegeneration. In this review, we describe microglial phenotypes and functions in the context of the young and ageing retina, and the mechanisms underlying changes in ageing. Additionally, we review microglia-mediated retinal neuroinflammation and discuss the mechanisms of microglial involvement in retinal neurodegenerative diseases.

## Introduction

The retina is an extension of the central nervous system (CNS). Retinal ganglion cells (RGCs) display the typical properties of CNS neurons, and their axons collectively form the optic nerve that connects the retina to the visual cortex in the brain. As a part of the CNS, the retina exhibits similarities to the brain in terms of anatomy, functionality, immunology, and pathology. Thus, the knowledge and insights obtained from retinal research are highly likely to apply to the brain, and vice versa.

Microglial cells are the resident macrophages of the CNS, including the retina, and they act as the first line and principle form of active immune defence. Microglia were long considered to be in a resting state in the healthy CNS. However, mounting evidence has shown that microglia are actively involved in the maintenance of the CNS. They are extremely sensitive to even subtle changes in the environment and therefore they are found to be constantly scavenging the CNS for damage, pathogens, and aggregates or debris. This sensitivity is achieved by the presence of microglial surface receptors which receive signals from surrounding neurons and the extracellular matrix that may indicate a homeostatic imbalance (Raivich et al., 1999; Nimmerjahn et al., 2005). In response to these signals, microglia instantly transform their phenotype from the ramified “standby” to the hyper-ramified and activated modes, and can even adapt into the amoeboid mode that enables them to quickly migrate to sites in danger (Deng et al., 2009). However, with ageing, the ability of microglia to maintain immune surveillance and tissue repair declines. Whilst young retinal microglia exhibit a prompt response to injury by rapidly polarizing their processes and migrating to the injury site, aged retinal microglia appear to be less dynamic with fewer processes (Damani et al., 2011). Aged microglia associate with an imbalanced polarization by producing and releasing pro-inflammatory factors that are heavily implicated in retinal neurodegenerative diseases, including glaucoma, age-related macular degeneration (AMD), and diabetic retinopathy (Guzman-Martinez et al., 2019; Table 1).

**TABLE 1:** Overview of the possible roles and behaviour of microglia in the young retina, ageing retina, and retinal neurodegenerative disease.

Young	Immune defence and homeostasis	• Surveillance of surrounding microenvironment
		• Phagocytic clearance of pathogens, apoptotic cells, and protein aggregates/debris
		• Tissue repair
Ageing	Senescence	• ↓ Ability for surveillance and immune defence
		• ↓ Phagocytic ability → accumulation of protein aggregates and toxic debris
		• ↓ Ability for tissue repair
Neuro-degenerative disease	Neuroprotection	• Facilitation of innate and adaptive immune responses
		• Special phenotypes:
		∘ Rod microglia
		∘ DAM
		• Promotion of regeneration
	Neurotoxicity	• Promotion of neuroinflammation → tissue damage → functional impairment
		• Production of pro-inflammatory cytokines and chemokines
		• Abnormal phagocytosis
		• e.g. involvement in pathogenesis of glaucoma, AMD, diabetic retinopathy

*DAM, disease-associated microglia; AMD, age-related macular degeneration.*

Microglia are regarded as the professional phagocytes of the CNS, including the retina (Khurana, 2002; Franco-Bocanegra et al., 2019). Microglia actively clear retinal myelin debris at injury sites to help remyelination. However, the phagocytic capacity of microglia declines with ageing, and the age-related increase in myelin debris further challenges microglia which are already senescing (Safaiyan et al., 2016; Shobin et al., 2017). This ageing process leads to a progressive accumulation of protein aggregates and myelin debris, which further contribute to neuroinflammation and retinal neurodegeneration (Table 1).

In this review, we firstly, delineate microglial morphology and phagocytosis in the healthy young retina and secondly, discuss changes of microglial phenotypes and phagocytosis in the ageing retina. We also focus on the role of microglia in retinal neuroinflammation and the mechanisms of microglial involvement in retinal neurodegenerative diseases.

## Microglia in the Healthy Adult Retina

### Microglial Migration and Distribution

**FIGURE 1:**
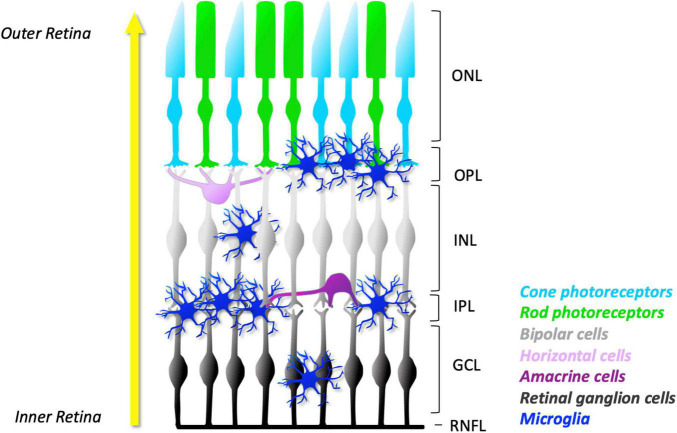
Schematic representation of microglial distribution in layers of normal mature retina. As development progresses, microglia migrate from the GCL and IPL towards the outer layers of the retina (yellow arrow). In the young retina, the number of microglia in their ramified appearance is found predominantly in the synaptic layers: the OPL and IPL. They can also be found in lesser numbers in the GCL and INL. However, no microglia are found in the ONL, a specialised microglial exclusion zone. The concentration of microglial processes in the plexiform layers facilitates frequent and dynamic contact with neuronal dendrites, axons, and synapses. The ramified morphology allows microglia to constantly extend and retract their processes. Together, these features facilitate constant surveillance of the surrounding microenvironment. ONL, outer nuclear layer; OPL, outer plexiform layer; INL, inner nuclear layer; IPL, inner plexiform layer; GCL, ganglion cell layer; RNFL, retinal nerve fibre layer.

Microglia are derived from the primitive yolk sac progenitors (Alliot et al., 1999). They migrate into the retina in a well-organised fashion, from the central to peripheral regions and the inner to outer retinal layers (Marín-Teva et al., 1998, 1999). The development of microglia in the retina plays a key role in shaping neuronal development and sculpting the initial population of early neurons to the organised subsets, i.e., photoreceptors, bipolar, amacrines, horizontal cells, and RGCs. With maturation, the five types of retinal neurons become arranged into three cellular layers and two synaptic layers with their specific input of light transmission (Figure 1). Photoreceptors, comprising the outer nuclear layer (ONL), relay visual information to the bipolar, amacrine, and horizontal neurons in the inner nuclear layer (INL) via the synapses in the outer plexiform layer (OPL). The visual information in the INL is further relayed to the RGC layer through the synapses with bipolar and amacrine cells in the inner plexiform layer (IPL) before being transmitted to the visual cortex in the brain (Figure 1).

Developing microglia migrate radially through the inner to outer retinal layers and predominately settle in the retinal synaptic layers (OPL and IPL) in a territorial and non-overlapping manner (Lee et al., 2008). While microglia can be also located in the retinal cellular layers (INL and RGC layer), they are notably devoid in the ONL, a specialized microglial exclusion zone (Santos et al., 2008; Figure 1). Microglia-specific locations in the retina may reflect their direct role in synaptic development and mature systems. Indeed, microglial processes in the plexiform layers allow them to frequently and dynamically contact dendritic, axonal, and synaptic compartments of neurons, being well-positioned to influence neuronal structure and therefore function.

### Microglial Morphology and Function

**FIGURE 2:**
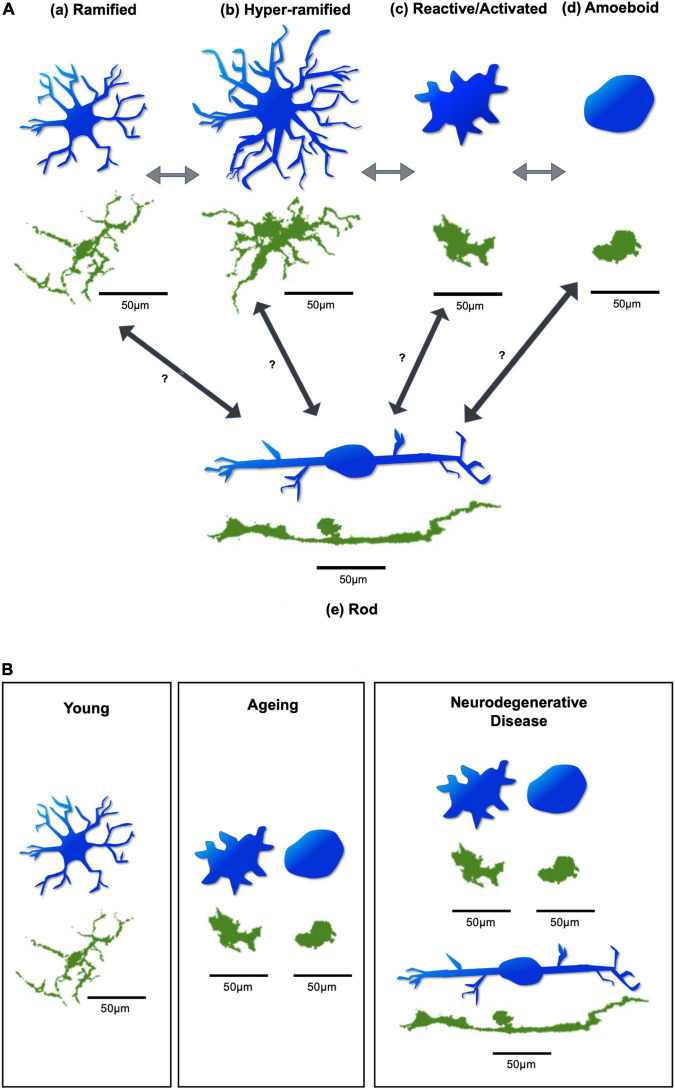
Diagram of microglial morphologies. (A) Microglial morphology varies based on activation state, in response to changing environmental conditions. Arrows demonstrate possible transitions between states. These transitions can occur in both directions, allowing microglia to switch back and forth between activation states. (a) Microglia under resting physiological conditions have a ramified appearance. (b) Disruption to environmental homeostasis leads them to elongate their processes and transiently exhibit a hyper-ramified state. (c) In response to considerable environmental damage, they rapidly adopt an activated morphology, with an increased soma size, and thicker, shorter processes. (d) Substantial insult leads them to adopt an amoeboid appearance, with complete retraction of processes, allowing directed motility and phagocytosis of target material. (e) Rod microglia, which are specifically associated with retinal neurodegeneration, exhibit a uniquely narrow and elongated morphology, with few processes. It is unclear which microglial activation state(s) give(s) rise to rod microglia. Figure based on Holloway (Holloway et al., 2019). (B) Different predominant morphologies and activation states of microglia may be found in the young, ageing, and neurodegenerative retina. Generally, ramified microglia may be predominantly found in the young retina. In the ageing retina, the reactive/activated and amoeboid microglia are predominantly found. In cases of retinal neurodegenerative disease, reactive/activated, amoeboid, and rod microglia are predominantly found. Blue microglia are illustrative representations to demonstrate morphological changes. Green microglia are from whole-mount retinal images of Iba-1 stained C57BL6 mice, obtained from the Cordeiro laboratory. Scale bar = 50 μm.

Microglia have diverse morphologies which are closely related to their activation status and functional phenotypes (Wolf et al., 2017). As the principal resident immune cells of the brain and retina, microglia are constantly engaged in the surveillance of their surrounding neural tissues. Whilst microglia are in “standby” mode, they are always ready to respond to or interfere with any homeostatic disturbances which may endanger neurons and threaten tissue integrity (Van Rossum and Hanisch, 2004). Under physiological (resting) conditions, microglia exhibit a ramified appearance with small and round somas, and many long and thin processes (Figure 2 and Table 2). This typical morphology allows microglia to constantly extend and retract their processes to scan and monitor their local microenvironment while their somatic positions maintain relatively stable (Nimmerjahn et al., 2005; Askew et al., 2017). *In vivo* two-photon brain imaging has shown that microglial processes are remarkably motile in the “resting” stage, continuously undergoing cycles of *de novo* formation and withdrawal within a time scale of minutes. In addition, their processes and protrusions directly contact neuronal cell bodies, astrocytes, and blood vessels, suggesting that in the healthy brain, microglia dynamically interact with other cortical elements to monitor neuronal well-being (Nimmerjahn et al., 2005). *In vivo* brain imaging has additionally demonstrated that the application of the ionotropic γ-aminobutyric acid (GABA) receptor blocker to change the level of neuronal activity evokes a significant increase in microglia volume sampling (Nimmerjahn et al., 2005). Like microglia in the brain, those in the retina also exhibit extensive dynamic behavior in *ex vivo* retinal whole-mounts (Lee et al., 2008; Liang et al., 2009).

**TABLE 2:** Microglial morphological parameters in various activation states.

Activation state	Soma shape	Soma size	Number of processes	Length of processes	Thickness of processes
Ramified	Round	**⋅**	**⋅⋅**	**⋅⋅**	**⋅**
Hyper-Ramified	Round/irregular/variable	**⋅⋅**	**⋅⋅⋅**	**⋅⋅⋅**	**⋅⋅**
Activated/reactive	Irregular/variable	**⋅⋅⋅**	**⋅⋅**	**⋅**	**⋅⋅⋅**
Amoeboid	Irregular/variable	**⋅⋅⋅⋅**	None	N/A	N/A
Rod	Elongated	?	**⋅**	?	?

*Dots represent relative values. Incomplete information on rod microglial morphological parameters due to limited data and difficulty in comparing their unique morphology.*

*Sources: Boche et al. (2013), Karperien et al. (2013), Chidlow et al. (2017), Davis et al. (2017), Holloway et al. (2019).*

Episodes of transient and localised microglial activation occur throughout life without necessarily being obvious or easily detected. However, relatively severe homeostatic alterations can cause excessive responses from microglia with significant morphological transformation. Some events may exceed critical limits of microglial responses, leading to disease pathogenesis. In such conditions, ramified microglia may become transiently hyper-ramified by elongating and increasing the number of their processes in response to initial environmental insult, and then quickly convert into the activated morphology if significant disruptions occur. This may occur by enlarging their somas and thickening and retracting their processes (Hanisch and Kettenmann, 2007; Figure 2 and Table 2). Activated microglia can further adapt into the amoeboid morphology when extensive damage occurs, by completely retracting their processes and further enlarging their motile somas. This allows them to quickly migrate to the sites of distress and phagocytose pathogens, dying neurons, or cell debris (Deng et al., 2009; Figure 2 and Table 2). There is another form of microglia, called rod-microglia, which has recently gained attention (Holloway et al., 2019). Rod-microglia, which appear as markedly narrow and elongated cells with a scanty cytoplasm and few processes (Figure 2 and Table 2), are thought to be associated with neuronal pathology in the CNS (Holloway et al., 2019). Rod-microglia have been reported in retinal degenerative disease models, such as glaucoma, a major cause of blindness and characterized by retinal ganglion cell (RGC) death and axonal degeneration. For instance, rod-microglia were found aligned with degenerating RGCs and their axons in mouse glaucoma models (de Hoz et al., 2013; Wang et al., 2014). Interestingly, these rod-microglia were only found in the affected eye of animals with unilateral retinal injury models, suggesting that this particular morphology of microglia in the retina may be associated with retinal neurodegeneration (de Hoz et al., 2013; Wang et al., 2014). Conversely, the association of rod-microglia with a protective role in retinal neurodegeneration was demonstrated when rod-microglia were found to be eliminating the degenerating RGC debris by phagocytosis in the retina of rats modelled with optic nerve transection (ONT) (Yuan et al., 2015). The morphologies of microglia described above can switch back and forth between activation states but it is unclear which microglial activation state(s) give(s) rise to rod microglia (Figure 2; Holloway et al., 2019). Moreover, a unique microglial phenotype, so-called disease-associated microglia (DAM), has recently been discovered (Keren-Shaul et al., 2017). DAM are characterised by the downregulation of homeostatic genes and the upregulation of genes involved in lysosomal, phagocytic, and lipid metabolism pathways, linked to Alzheimer’s disease (AD) and other neurodegenerative conditions. Their activation requires TREM2 (triggering receptor expressed on myeloid cells 2) (Keren-Shaul et al., 2017). Identified as phagocytic cells, DAM have an activated morphology, and have been found to colocalise with amyloid-beta (Aβ) plaques (Keren-Shaul et al., 2017). The DAM phenotype, as a common signature of microglial response to CNS pathology, may play a protective role in neurodegeneration (Deczkowska et al., 2018). By profiling the mouse retinal microglia across development, a recent study has demonstrated that microglia in the postnatal retina highly express disease-associated genes (DAM), which are similar to microglia in ageing, disease, and in developmental white matter (Anderson et al., 2019). In addition, this study identified developmental apoptosis as a major driver of the DAM-related profile in the postnatal retina (Anderson et al., 2019). DAM were also found in the adult retina in a transgenic mouse model of glaucoma (DBA/2J). By analyzing the transcriptome of retinal microglia with age, the authors found that DAM cells had a significant increase in the degenerative retina at 10months old compared to the pre-degenerative retina at 5 months old. Additionally, the shift to the DAM profile during the course of DBA/2J was similar to that in brain neurodegeneration, with the upregulation of genes related to phagocytosis and lipid metabolism and downregulation of select homeostatic genes (Bosco et al., 2019). Although it was unclear whether DAM are detrimental or protective in DBA/2J, the authors have found a more pronounced DAM gene expression signature after neuroprotective complement inhibition, suggesting a potential protective role (Bosco et al., 2019).

The transformation of ramified microglia into their activated mode can be triggered by signals reporting homeostatic disturbance. Such signals can be any abnormal appearance, unusual concentration, or altered features of soluble and insoluble factors indicating local danger or damage (Hanisch, 2002; Streit, 2002). In a retinal degeneration model of *rd* mice, some chemokines were demonstrated to initiate microglial activation (Zeng et al., 2005). By identifying sequential events and factors associated with microglial activation, migration, and cytotoxicity during retinal degeneration, this study found that the expression of the chemokine mRNA transcripts: MCP-1, MCP-3, MIP-1alpha, MIP-1beta, and RANTES, was first noted at P8, reaching a peak at P12, well before the initiation and peak of microglial migration. This suggests the role of these chemokines in microglial activation and trafficking of the retinal microglia to the degenerating photoreceptor layer. It has been hypothesized that in the *rd* retina, the gene defect in beta-PDE causes the initial injury to the photoreceptors which produce chemokines to activate and recruit retinal microglia to the outer retinal layers (Zeng et al., 2005). The primary death of photoreceptors triggering microglial activation and their migration to the outer retina has also been observed in retinitis pigmentosa (RP) mice (Noailles et al., 2014). Endogenous photoreceptor proteins have been reported to trigger microglial activation via toll-like receptor 4 (TLR4) (Kohno et al., 2013). Early alarm signals from retinal degeneration might initiate TLR-dependent activation of microglia (Langmann, 2007). In the retina of retinoschisin-deficient mice, early microglial TLR4 induction has been detected with a strong up-regulation of early microglial activation-related transcription (Gehrig et al., 2007). TLR-dependent pathways in initiating microglial activation have also been observed in other models of neuronal injury, including spinal nerve transection (Lehnardt et al., 2003). Microglia can sense these warning signals via their surface receptors and ion channels, such as receptors for cytokines, chemokines, complement fragments, immunoglobulins, and inflammatory stimuli (Raivich et al., 1999; Nimmerjahn et al., 2005). As such, endangered or stressed neurons emit signals of cytosolic or membrane origin, which cause gradual transformation of microglia. Such signals can be subtle or even remote (Streit, 2002). ATP concentrations rising beyond a critical threshold could signal abnormal firing activity and cellular disintegration in the retina and brain (Hide et al., 2000; Honda et al., 2001; Damani et al., 2011). Additionally, excessive neurotransmission may result in a high state of microglial activity (Hide et al., 2000; Honda et al., 2001; Inoue, 2002). Microglia will subsequently respond with supportive and protective activities, safeguarding the innate defence mechanisms, or assisting in the adaptive (i.e., antigen-specific) immune reactions (Van Rossum and Hanisch, 2004). For example, microglia may facilitate the removal of excitatory amino acids (Nakajima et al., 2001; Vallat-Decouvelaere et al., 2003), the expression of neurotrophic factors (Elkabes et al., 1996; Hanisch, 2002), or the stimulation of other cells, e.g., Müller cells to release neurotrophic factors (Harada et al., 2003; Wang et al., 2011). However, microglial activation can also be harmful, producing pro-inflammatory cytokines/chemokines, promoting neuroinflammatory responses, and causing tissue damage (Mantovani et al., 2004; Orihuela et al., 2016).

### Microglial Phagocytosis

One of the important physiological functions of microglia is to clear cellular debris and dead cells, the so-called process of “phagocytosis.” Microglial phagocytosis starts initially during the development of the CNS, when excessive neurons and synapses need to be removed in a process known as “synaptic pruning” (Paolicelli et al., 2011). In the mature brain and retina, highly motile microglia constantly change their morphology to adapt to the environment and transform into macrophage-like amoeboid shapes in response to alarm signals indicating tissue damage and debris accumulation. This amoeboid morphology allows microglia “directed motility” or “chemotactic motility” toward the source of injury (Khurana, 2002; Franco-Bocanegra et al., 2019). It is thought to be driven by complex molecular machinery, directing the actin cytoskeleton to the site of injury (Khurana, 2002; Franco-Bocanegra et al., 2019).

Phagocytosis occurs in several steps characterised by recognition (“find-me”), phagosome formation (“eat-me”), and ingestion (“digest-me”) (Sierra et al., 2013; Gabandé-Rodríguez et al., 2020). The phagocytic activity relies on specific receptors expressed on the macrophagic/microglial cell surface and downstream signaling pathways to direct the phagocytes toward the “eat-me” targets (pathogens, dead cells, or protein aggregates). The process is initiated by the activation of several membrane receptors that in general, can be categorized into two distinctive types, one having a high-affinity binding to foreign microbial pathogens, such as TLRs, and another mainly recognizing apoptotic cells exposing phosphatidylserine (PS), such as TREM-2 (Takahashi et al., 2005; Mazaheri et al., 2017) and TAM (Tyro3, Axl and Mer) receptors (Stefanova et al., 2011; Venezia et al., 2017; Gabandé-Rodríguez et al., 2020). In addition to their roles in the recognition of pathogenic or dead cell-associated targets, TLRs also recognize danger-associated molecular patterns, such as deposited Aβ fibrils and α-synuclein (Marsh et al., 2009; Hanke and Kielian, 2011). TREM-2 can signal for the internalisation of protein aggregates, such as Aβ (Cho et al., 2014; Han et al., 2017; Krasemann et al., 2017). In the end, degradation of the engulfed target or the apoptotic cell takes place once the phagosome becomes mature and fuses with lysosomes within the phagocyte, a process known as corpse processing or phagosome maturation (“digest-me”) (Sierra et al., 2013; Gabandé-Rodríguez et al., 2020).

## Microglia in the Ageing Retina

### Microglial Phenotype Changes in Ageing

Microglia are long-living cells with prolonged residence in the brain and retina, and senescent alterations may compromise their physiological roles in immune surveillance and tissue repair. In the young healthy retina as mentioned above, ramified microglia exhibit long branching processes, and rapid and constitutive motility of their processes enables the cells to effectively survey the extracellular milieu in the surroundings. Upon local injury, microglia promptly polarize their processes and migrate in the direction of the injury site. However, with ageing, these featured phenotypes of retinal microglia may progressively change. Studies on microglial ageing in the retina have shown that, compared to young (3–4 months old) mice, microglia in aged (18–24 months old) mice had fewer branches with shorter total process lengths indicating significantly smaller ramified dendritic arbours (Figure 2). Despite this, they still maintained their ramified morphology in a “tiled” distribution in the IPL and OPL (Damani et al., 2011). Aged microglia also exhibited slower dynamic movement of their processes than their younger counterparts (Damani et al., 2011). Similar observations on aged microglia have also been reported in the cortex and hippocampus (Hefendehl et al., 2014; Davies et al., 2017). These ageing changes in both morphology and constitutive motility may undermine microglial ability to survey and continuously interact with their environment. Interestingly, the densities of microglia soma in the IPL and OPL appeared to be slightly but significantly greater in the aged retina compared to the young retina (Damani et al., 2011). However, instead of an increase in soma densities and a decrease in process lengths, a more recent study found that ageing mice had significantly increased microglial soma area and vertical processes in OPL, IPL, and NFL-GCL layers (Fernández-Albarral et al., 2021a). These results imply that with ageing, microglia may shift their morphology and activity status to accommodate the ageing process. Indeed, increased microglial activation in the retina has been observed in old (20-month-old) mice compared to young (3-month-old) mice, along with increased complement activation (Chen et al., 2010). This study investigated microglia in retinal whole-mounts by dual staining using Iba-1 (microglial marker) and Isolectin B4 (IB4, a marker for activated microglia). Whilst no IB4^+^ microglia were observed in the young retina, intense IB4^+^ staining was found in the old retina in Iba-1^+^ microglia in the superficial layer and INL which appeared small and round or amoebic with short dendrites (Chen et al., 2010). In addition, the ageing retina has been associated with dysregulation and changes in their genetic profile including those genes linked to local inflammatory responses and phagocytosis (Chen et al., 2010; Fernández-Albarral et al., 2021a).

Whilst in young animals, few or no microglia were shown to be located in the outer retina (a microglial exclusion zone), aged animals had significantly greater numbers of microglia in either the subretinal space or with their processes oriented toward the subretinal space (Xu et al., 2008; Damani et al., 2011). The subretinal microglia exhibited an activated phenotype with a large cell body and short dendrites, associated with autofluorescent lipofuscin granules (Xu et al., 2008). It has been suggested that the subretinal presentation of microglia may be attributed to age-related changes in microglial behaviour in response to extracellular signals (Wynne et al., 2010) as the relocation of microglia to the outer retina was also seen in mice deficient in chemokines and chemokine receptors (Combadière et al., 2007).

Microglial responses to injury have also been shown to alter with ageing in mice. For example, when being exposed to extracellular ATP (an injury-associated signal), young microglia were found to exhibit instant dynamic responses by becoming more ramified and increasing their motility. However, aged microglia appeared to be less ramified and dynamic (Damani et al., 2011). These age-dependent dynamic behaviours were also evident in response to laser-induced focal retina injury, where aged microglia not only migrated to the injury site more slowly, but also exhibited a slower disaggregation from the injury site compared to their young counterparts (Damani et al., 2011). The age-related changes of microglial response to injury have also been seen in the brain, correlating to increased neuronal damage and functional loss (Conde and Streit, 2006; Wasserman and Schlichter, 2008; Ritzel et al., 2019). This suggests that aged microglia have a decreased capacity for tissue repair.

Microglial ageing in the human retina has been less studied than animal models. However, a recent study has demonstrated that, similarly to the aged retina in animal models, the human retina also showed an increased number of microglia during ageing (Yi et al., 2020). This study provided a comprehensive transcriptomic atlas based on 119,520 single cells of the human retina covering different ages. It was found that the proportion of microglia was remarkably increased in the aged samples, consistent with the up-regulated genes, which were enriched in response to hypoxia and microglial activation (Yi et al., 2020). Neuroinflammation and microglial activation have been implicated in retinal ageing and neurodegenerative diseases, and increased pro-inflammatory factors in the aged retina may indicate increased microglial involvement. 7-ketocholesterol (7KCh) is a cholesterol oxidation product localized to the outer retina with pro-inflammatory and cytotoxic properties in AMD (Rodriguez et al., 2014). Quantification of 7KCh showed that the levels of 7KCh were significantly higher in older human donors (Rodriguez et al., 2014). By exploring the potential relationship between 7KCh and microglia, another study demonstrated an age-dependent accumulation of 7KCh in subretinal microglia of mice. 7KCh was shown to exert a prominent dose-dependent chemoattractive effect on retinal microglia *in vivo* and *in vitro* (Indaram et al., 2015). In addition, subretinal injection of 7KCh induced a remarkable increase in the number of Iba1 positive microglia into the outer retina (Indaram et al., 2015). Taken together, it is possible that higher levels of 7KCh found in the aged human retina may recruit microglia into the outer retina and stimulate microglial internalization of 7KCh as that occurred in animal models.

### Microglial Phagocytosis Changes in Ageing

In order to maintain homeostasis and tissue repair, microglial phagocytosis is essential for the clearance of cellular debris, abnormal proteins, and pathogens from the CNS, including the retina (Li, 2013). However, the phagocytic capabilities of microglia tend to be compromised with ageing, resulting in a more toxic CNS environment, and leading to neuronal degeneration (Li, 2013). Studies on the uptake of neurotoxic materials by cultured microglia have provided evidence of an age-dependent decline in microglial phagocytosis. For instance, microglia from aged mice appeared to have a reduced capacity to internalise Aβ compared to young mice (Njie et al., 2012). The age-related loss in microglial ability to phagocytose Aβ fibrils has also been demonstrated to be associated with altered expression of Aβ interacting protein (CD36) (Floden and Combs, 2011). Similar findings have been seen with neonatal microglia cultured for an extended period compared to those cultured over a shorter period of time (Caldeira et al., 2017). The extended period microglia showed lower efficiency in phagocytosis of Aβ. This was associated with the dysregulation of phagocytosis-related receptors or proteins, including increased expression of CD33 and reduced levels of TREM2 and MFG-E8 (milk fat globule-EGF factor 8 protein) (Caldeira et al., 2017). Aβ deposits in the retina have the potential to be a targeted biomarker for early AD diagnosis and disease progression. The natural compound curcumin binds to Aβ with a high degree of specificity and thus, retinal imaging of fluorescent curcumin has been explored in AD clinical trials and shown promising results (Koronyo et al., 2017; Dumitrascu et al., 2020; Ngolab et al., 2021). A positive correlation between retinal curcumin-positive spots and brain amyloid has been observed in patients, suggesting that retinal curcumin imaging holds potential for the detection of preclinical AD and the progression of dementia (Ngolab et al., 2021). Regarding the treatment and clearance of amyloid deposits, a recent study has co-cultured brain slices from aged AD mice with young WT mice and has shown that the phagocytic ability of aged microglia can be restored through factors secreted from young microglia, resulting in increased amyloid plaque clearance. Also, exposing aged microglia to conditioned media of young microglia was sufficient to induce microglial proliferation and reduce amyloid plaque size (Daria et al., 2017). Age-related decline in microglial phagocytic capacity has also been observed in the uptake of α-synuclein, a presynaptic neuronal protein (Koronyo et al., 2017) linked to Parkinson’s disease (PD) pathology. By comparison of microglia isolated from adult mice to neonatal mice, researchers have shown that adult microglia exhibited less efficiency in phagocytosis of free or exosome-associated oligomeric α-synuclein compared to microglia from young mice (Bliederhaeuser et al., 2016).

Instead of assessing microglial phagocytosis by *ex vivo* culture systems which have lost tissue integrity, a recent study has used immunohistochemistry and stereology to identify and quantify activated and phagocytic microglia in rhesus monkeys (Shobin et al., 2017). They found that microglia became activated with age and had an increased phagocytic phenotype in regions of white matter. The age-related increase in phagocytic activation of microglia has been associated with accumulating myelin pathology (Shobin et al., 2017). Myelin phagocytosis is the pathological hallmark of multiple sclerosis (MS), but it remains unclear what triggers resident microglia and infiltrating macrophages to start phagocytosing myelin. To investigate this, researchers have isolated myelin from brain samples of MS patient donors and normal controls, and assessed its uptake by primary human microglia and THP-1 macrophages. The authors found that both microglia and macrophages appeared to be more efficient at phagocytosing MS-derived myelin than control myelin, and microglia from aged donors had an increased myelin uptake compared to young donors, indicating that changes in myelin composition during ageing may trigger phagocytosis and contribute to MS pathology (Hendrickx et al., 2014).

Myelin breakdown has significant consequences for axonal health and conduction velocity (Saab and Nave, 2017). Microglia actively clear myelin debris at injury sites to help remyelination. However, an age-related steady increase in myelin debris would overwhelm microglial phagocytosis, leading to myelin accumulation and accelerated neural degeneration. Using electron microscopy to analyse the white matter of ageing mice, progressive accumulation of multilamellar myelin fragments has been demonstrated with age (Safaiyan et al., 2016). An increased number of microglia in the white matter was also reported in aged mice (Poliani et al., 2015). To determine whether microglia were responsible for the uptake of myelin fragments, researchers have used immunohistochemistry to localise microglia, myelin basic protein (MBP), and proteolipid protein (PLP) in white matter (Safaiyan et al., 2016). An increased number of Iba-1^+^ microglia were found to be in contact with myelin fragments and also phagocytosing them. There was also an age-related increase in markers for endosomes and lysosomes in microglia, including scavenger receptor (CD68) and lysosomal-associated membrane protein 1 (Lamp1), along with increased surface markers for myelin phagocytosis (Mac-2) in the white matter (Safaiyan et al., 2016). Furthermore, a steady accumulation of lipofuscin granules, one of the most specific biomarkers for ageing, was observed in microglia with increased age, and also associated with the uptake of myelin debris. Taken together, this suggests that microglia are not only actively involved in the clearance of myelin, but with ageing, this process is also associated with the accumulation of undegradable lysosomal aggregates, which may contribute to microglial senescence and immune dysfunction in the aged CNS (Safaiyan et al., 2016).

## Retinal Microglia in Degenerative Eye Diseases

Degeneration within the retina is a common pathological mechanism in various neurodegenerative diseases, involving microglia-associated neuroinflammation (Karlstetter et al., 2015; Ramirez et al., 2017; Silverman and Wong, 2018; Rashid et al., 2019). Neurodegeneration in the retina occurs not only in eye diseases, e.g., glaucoma, AMD, and DR, but also in neurological disorders, e.g., AD, PD, and MS. There is currently no efficient treatment for retinal neurodegeneration. In this section, we focus only on retinal microglia in neurodegenerative eye diseases. Imbalanced microglial activation is heavily implicated in retinal neurodegeneration (Karlstetter et al., 2015; Ramirez et al., 2017; Silverman and Wong, 2018). The activated microglia can release pro-inflammatory cytokines which can aggravate and propagate neuroinflammation, thereby promoting neuronal degeneration and eventually functional impairment (Rashid et al., 2019). Microglial activation in these diseases has been related to toxic protein aggregates, abnormal phagocytosis, and neuronal degeneration (Karlstetter et al., 2015; Ramirez et al., 2017; Silverman and Wong, 2018).

### Glaucoma

Glaucoma is the leading cause of irreversible blindness, predicted to affect approximately 112 million people by 2040 (Rashid et al., 2019; Allison et al., 2020). It is a multifactorial disease with several causative factors, such as myopia, intraocular pressure (IOP), and optic nerve blood supply. Several sub-types, such as primary open angle, primary angle closure, secondary open angle, secondary angle closure, and normal tension glaucoma have been identified (McMonnies, 2017; Dietze et al., 2022). Generally, these types of glaucoma can be characterized by RGCs and their axonal death, leading to vision loss (Davis et al., 2016; Rashid et al., 2019). The underlying mechanisms are not fully understood although ocular hypertension (OHT) has, for a long time, been correlated to RGC death and vision loss (Rashid et al., 2019). Neuroinflammation induced by microglial activation has been observed since the 1970s and has become increasingly recognised as a key mechanism in the pathogenesis of glaucoma (Vrabec, 1975; Rashid et al., 2019).

There is evidence to show that microglia-associated inflammation occurs throughout glaucoma progression. In a mouse model of glaucoma (induced OHT), distinctive changes in expression of M1 and M2 associated factors were noticeable from the first day (Fernández-Albarral et al., 2021b). From day 1 post OHT, anti-inflammatory factors such as IL-4, VEGF, and BDNF were all significantly increased in expression. Meanwhile, pro-inflammatory factors such as IL1-ß, were decreased in expression (Fernández-Albarral et al., 2021b). This suggests there may be initial M2-associated activation in an attempt to reduce inflammation, repair damage, and recover a healthy microenvironment. Additionally, levels of microglial activation regulator, Cx3CL1, were significantly increased only on the first day post OHT, then this decreased to a level that was significantly lower than healthy controls from day 3 onwards (Fernández-Albarral et al., 2021b). Interestingly, the number of RGCs was also significantly reduced from 3 days post OHT. A previous investigation that used the same glaucoma model also showed retinal microglia activation peaking at 3 days post OHT, with increased microglia cell density, but enlarged cell bodies and fewer dendrites (Ramírez et al., 2020). These amoeboid microglia were found to lack expression of P2RY12, a common marker of resting microglia associated with the M2 phenotype (Ramírez et al., 2020). This suggests that in this model, up to day 3, there may be M2 activation and fractalkines may have a chemoattractant effect on the surveillant processes of microglia and by day 3, microglia may start their process of eliminating deleterious cell fragments. Furthermore, in another model using laser induced OHT rats, at Day 14 post OHT, amoeboid microglia were found to be co-localised with major histocompatibility complex (MHC) II (de Hoz et al., 2018), an M1 marker associated with toxic cell fragments and pathogens (Charles et al., 2001). A similar finding of MHC II+ co-localisation with microglia was also found to coincide with OHT-induced axonal and RGC loss (Chidlow et al., 2016). de Hoz et al. (2018) suggest that MHC II may influence ramified surveillant cells to transform into the amoeboid phagocytic microglia. Additionally, in patients with mild to advanced stages of glaucoma, the expression of proinflammatory or M1 associated factors, such as TNF-alpha and TGF-beta, and microglial densities are significantly higher compared to healthy volunteers (Yuan and Neufeld, 2001). These studies indicate an initial neuroprotective microglial response, to produce neurotrophic factors with efforts to remove deleterious cell debris, that may, when prolonged, transform into a pathological inflammatory response. Although the exact mechanisms and orders of these events are still unknown, this suggests that microglia-associated immunomodulatory changes may occur from the early stages of glaucoma, and is similar to other CNS neurodegenerative disease process, as described earlier.

### Age-Related Macular Degeneration

Age-related macular degeneration (AMD) is the leading cause of blindness in the elderly population worldwide (Miller et al., 2021). In 2020, ∼70 million of those in the EU were living with AMD and this is predicted to increase by at least 15% by 2050 with the rise in average life expectancy (Li et al., 2020). AMD affects the central macular region of the retina, resulting in abnormalities of the structural and molecular composition of photoreceptor (PR), retinal pigment epithelium (RPE), Bruch’s membrane, and choriocapillaris (Chirco et al., 2016). Early AMD is characterised by yellow drusen, which are composed of proteins and lipids such as lipofuscin and Aβ peptides (Wu et al., 2021), that lie within or beneath the RPE (Silverman and Wong, 2018), and RPE and photoreceptor degeneration (Borucki et al., 2020). The disease may progress to later stages, broadly defined by two types: dry AMD advancing to geographic atrophy (GA) (Waugh et al., 2018) or wet AMD characterized by choroidal neovascularisation (CNV) (Silverman and Wong, 2018). These pathophysiological features of AMD have been correlated with microglia-associated inflammation.

The characteristics of microglia have been investigated in the context of drusen-associated pathogenesis using Aβ injections as Aβ is a component of drusens and has been associated with inflammation (Wu et al., 2021). Whole retinal mounts of C57 mice administered with Aβ by subretinal injections (to model drusen) were found to have more retinal cells expressing Iba-1, exhibiting amoeboid morphologies by the 3^rd^ day post administration, with almost ×3 greater cell diameter compared to healthy controls administered with PBS (Wu et al., 2021). There was also higher expression of pro-inflammatory cytokines including translocator protein (TPSO), COX-2, and IL-1β, and this was also observed in *in vitro* primary microglial cell cultures when modelled for drusen with Aβ administration (Wu et al., 2021). When these primary cultures were co-cultured with photoreceptor-like cells (661W), there was a significant reduction of 661W cells and significantly greater caspase 3 (apoptosis assay), compared to the control cultures (Wu et al., 2021). This suggests that early microglial activation with morphological and transcriptional changes may be an initial response to drusen-associated insult, in efforts to remove deleterious debris. In addition, drusens and AMD have been associated with polymorphisms in CX3CR1 which are only expressed by microglial cells in the entire retina. For instance, 12 month old CXCR1^–/–^ mice were found with subretinal drusen deposits co-localised with microglia whilst age-matched CXCR1^+/+^ mice did not (Combadière et al., 2007). Additionally, such subretinal microglial accumulation seen in the CXCR1^–/–^ mice was associated with retinal degeneration including photoreceptor loss (Combadière et al., 2007). This further supports the role microglia and inflammatory mediators may play in AMD drusen pathology.

Microglia-associated inflammation is also implicated in the pathogenesis of CNV formation. For example, microglial transforming growth factor (TGF) β plays a major role in the development and maintenance of neurons and retinal blood vessels (Ma et al., 2019). Although TGFβ has been associated with AMD and CNV pathologies, its exact role and mechanism of action in disease pathogenesis is still uncertain (Tosi et al., 2018). For instance, increased expression of TGFβ has been found in the retinal endothelial cells in CNV animal models and systemically induced TGFβ inhibition resulted in reduced CNVs, suggesting TGFβ may play a role in inducing CNV pathology (Tosi et al., 2018). In contrast, tamoxifen-induced elimination of TGFβ receptors on retinal microglia resulted in greater CNV growth, suggesting that the suppression of microglial TGFβ signaling may contribute towards CNV pathogenesis (Ma et al., 2019). Additionally, flat-mount retina studies demonstrated ramified microglia in normal conditions, but 2–5 days post tamoxifen administration, microglia had much fewer and thicker processes, resembling the activated morphotype (Ma et al., 2019). By 3–10 weeks post-tamoxifen, they appeared “elongated” and aligned along the retinal vasculature. Despite the general consensus of amoeboid microglia being the phagocytic morphotype, both activated (Namekata et al., 2019) and rod (Yuan et al., 2015) microglia have also been reported in association with phagocytic functions (Ma et al., 2019). Interestingly, post-mortem histology assessment of retinae from CNV donors showed amoeboid microglia with rhodopsin labelled inclusions located in the ONL where there was undergoing rod photoreceptor degeneration (Gupta et al., 2003). Thus, the authors suggest that microglia phagocytose rod cells as a result of microglial activation, triggered by rod photoreceptor degeneration (Gupta et al., 2003). To our knowledge, there are no studies that investigate the changes in the characteristics of retinal microglia in relation to phagocytic functions that may be induced by CNV-associated changes to TGFβ expression. There are, however, studies that have found that TGFβ is associated with phagocytosis by retinal cells including microglia (Sheu et al., 1994; Bialas and Stevens, 2013; Sharma et al., 2014; Silverman and Wong, 2018). Bialas and Steven have observed that Tgfbr2^–/–^ animals had a deficient expression of complement protein C1q (which has been associated with AMD), and both Tgfbr2^–/–^ and C1q deficient (C1qa^–/–^) animals had reduced rates of RGC phagocytosis compared to age-matched controls (Bialas and Stevens, 2013). Future investigations could explore if similar observations in relation to protein expression, microglial morphologies, and microglial phagocytosis may be seen in the context of CNV formation.

Additionally, there are senescent molecular changes that may trigger microglial activation ultimately resulting in AMD-associated structural changes. Immunohistological observation of 7-ketocholesterol (7-KCh), a derivative of cholesterol associated with pro-inflammatory responses, showed greater expression in 24 month old mice than in 2 month old mice, and the 7-KCh expression was also co-localised with GFP-positive microglia (Indaram et al., 2015). In vitro observations showed that microglia transition from ramified to amoeboid with the administration of 7-KCh. Additionally, quantitative reverse transcription polymerase chain reaction (qRT PCR) analysis showed 7-KCh-induced reduction of neurotrophic factors, e.g., BDNF and NGF, and an increase in angiogenic factors, e.g., VEGF (Indaram et al., 2015). Finally, CNV was observed in 3–4 month old mice, 7 days post-transplantation of microglia which had exposure to 7-KCh, whilst those that had the administration of microglia exposed to control media did not (Indaram et al., 2015). This further supports that senescent changes in the expression of proteins are capable of polarising microglia from the M2 to M1 pro-inflammatory type as evidenced by morphological and transcriptional observations and in turn resulting in CNVs.

Photoreceptor degeneration, a pathological feature of late-stage AMD, has been associated with changes in the metabolic process of PRs (Cheng et al., 2021). For this investigation, microglia were observed from flat mounted retinas. Flat mounted observations may be advantageous especially with advanced imaging equipment and image processing software as it may allow analysis of the retina as a whole with the additional option to observe across extended depths of fields through the different layers of the retina. Whilst cross sectional observations of the retina may allow a more refined observation of the distinctive layers of the retina, it typically allows observation of one slice per time with limited location and information. Although it would be possible to create a montage of several slices to observe a more comprehensive view of the retina, this would require a more complex and lengthy process. In this study, the removal of tuberous sclerosis complex (TSC) proteins, TSC1 and TSC2, was performed on mice to mimic such altered metabolic profiles of PRs (Cheng et al., 2021). Whilst TSC1^–/–^ mice were found with more ramified retinal microglia from flat-mount observations, TSC2^–/–^ mice were found with more amoeboid which also co-localised with MHCII (Cheng et al., 2021). Interestingly, whilst both groups of mice were found to develop GA, TSC2^–/–^ mice developed changes in a gradually enlarging circular pattern which is typical of that seen in AMD patients with GA (Cheng et al., 2021). Additionally, TSC2^–/–^ mice were found with reduced expression of complement protein C3, compared to TSC2^+/+^ whilst TSC2^–/–^ also had reduced phagocytic activity of RPE compared to controls and TSC1^–/–^ (Cheng et al., 2021). Although C3 has been associated with microglial phagocytosis (Borucki et al., 2020), this particular observation in this study was not made exclusively in microglial phagocytosis (Cheng et al., 2021). Overall, TSC-mediated clinically translatable AMD GA pathology may be associated with observations of retinal amoeboid microglia, reduced C3 expression, and reduced ability to clear RPE debris by phagocytosis. Despite this, there has also been contradicting evidence whereby both amoeboid microglia and increased expression of C3 have been reported in the ageing retina (Chen et al., 2010). Since age is a major risk factor for AMD, it has also been reported that an increase in microglial C3 may contribute to AMD pathology by modification of the phagocytic functions essential for synaptic pruning and removal of debris including RPEs, PRs, and amyloid deposits (Ma and Wong, 2016). There has also been a clinical trial that looked at intravitreal administration of pegcetacoplan, a C3 inhibitor, which slowed down the rate of GA progression, although a large proportion of patients who took the drug were reported to have developed new CNVs (Liao et al., 2020).

### Diabetic Retinopathy

Diabetic retinopathy (DR) is the most common cause of visual disease seen in patients with diabetes mellitus (Karlstetter et al., 2015). DR is characterised by inflammation, elevated vascular endothelial growth factors (VEGFs), and neuronal, vascular, and blood-retinal-barrier (BRB) damage, with proliferative disease and macular oedema commonly treated with intravitreally administered anti-VEGFs (Kinuthia et al., 2020; Xie et al., 2021). The major role that microglia-associated neuroinflammation may play throughout the pathogenesis of DR has become increasingly evident (Karlstetter et al., 2015; Kinuthia et al., 2020), whilst there has also been emerging evidence of deficiencies in therapeutic success with anti-VEGFs (Kinuthia et al., 2020; Wallsh and Gallemore, 2021).

There are early microglial changes that occur in DR pathogenesis which may also be prolonged through to later stages of DR. Using streptozotocin (STZ), a commonly used antibiotic, diabetes can be induced in animal models by causing toxicity to β cells in the pancreas that normally produce insulin (Graham et al., 2011). Although no difference in microglial density has been found, significantly more amoeboid and hypertrophic microglia have been found in rat retinal cross-sections, following 4, 8, and 12 weeks of intraperitoneal administration of STZ, compared to healthy controls (Chen et al., 2015). Interestingly, the authors included both hypertrophic and amoeboid microglia as a part of their observations in “activated” microglia. Furthermore, by 12 weeks post STZ-administration, microglial levels in the IPL decreased, whilst they increased in the RNFL and GCL (Chen et al., 2015). This re-organisation may be due to the gradual thinning of the IPL or attempts at the removal of deleterious RGCs in the RNFL/GCL (Chen et al., 2015). Similar occurrences have been reported in a different model of diabetes and DR (Goto Kakizaki rats, GK) in hyperglycaemic GK rats from 2 to 12 months of age. There were significant increases in Iba-1 positive microglia in the inner layers of the retina which appeared amoeboid by 12 months (Omri et al., 2011). Similar observations have been seen in human tissue with hypertrophic microglia present across all retinal layers in the earlier stages of DR (non- and pre-proliferative DR) (Zeng, 2008). Amoeboid microglia have been observed around the retinovascular exudates in the later stages of DR (proliferative DR) (Zeng, 2008). This could imply that the phagocytic amoeboid morphotype is more actively involved in later stages of clinical DR, however, further validation and a greater sample size is necessary. In terms of microglia-associated factors, there have been reports showing early changes in expression of CD11b, C1r, IL-1β, IL-6, and TNF-α, in the context of DR-associated pathologies e.g., vascular permeability, neuronal function, and neovascularisation (Rangasamy et al., 2014; Karlstetter et al., 2015; Madeira et al., 2015; Kinuthia et al., 2020; Li et al., 2021; Xie et al., 2021; Yun, 2021). Such changes in expression have also been correlated with amoeboid and activated microglia, microglial ‘activation’, apoptosis, and phagocytosis (Rangasamy et al., 2014; Karlstetter et al., 2015; Madeira et al., 2015; Kinuthia et al., 2020; Li et al., 2021; Xie et al., 2021; Yun, 2021). Primary retinal cell cultures subjected to hypoxic conditions (as a model of DR), show increased levels of C1r, a marker of phagocytosis, and CD11b, a marker of microglial activation, by 135.5 and 170%, respectively, compared to normoxic conditions (Xie et al., 2021). In the same study, STZ-administered rats showed amoeboid retinal microglia, compared to ramified microglia in healthy retinas (Xie et al., 2021). By 8 weeks post STZ-administration, these amoeboid microglia were found phagocytosing endothelial cells, causing increased acellular capillaries and vascular permeability, both distinctive features of DR (Xie et al., 2021). There are, however, contradictory reports (which could be due to different animal models used) where GK rats were found with retinal microglia that were mostly ramified and some hyper-ramified (Hachana et al., 2020), even though they had higher expression of Iba1, CD11, and VEGF compared to age-matched controls. There was also a greater number of blood vessels and greater vessel tortuosity that progressively developed, indicating DR-related endothelial abnormalities (Hachana et al., 2020). The initial elevation in the expression of cytokines (IL-1β, IL-6, and TNF-α) may induce microglia to release complement proteins in an attempt to restore physiological conditions in DR (Chen and Xu, 2015; Xu and Chen, 2017; Kinuthia et al., 2020). However, as DR progresses and hyperglycaemic conditions are prolonged, mechanisms involving protein kinase C (PKC) are activated, resulting in the build-up of advanced glycation end-products (AGEs) (Kinuthia et al., 2020). This in turn can disrupt the complement system, thereby impairing the ability to restore health and promoting vascular and PR-associated DR pathologies (Inafuku et al., 2018). In addition, as the accumulated AGEs interact with their receptors, this encourages pro-inflammatory M1 microglia which contributes further to the release of inflammatory cytokines and a neurotoxic environment (Kinuthia et al., 2020).

Previous literature has reported that up to 50% of patients on anti-VEGF therapies have had insignificant levels of or a complete lack of improvement (Kinuthia et al., 2020; Wallsh and Gallemore, 2021). This resistance to anti-VEGFs has recently been found to also be associated with microglia-associated inflammatory responses. Hence, anti-VEGF treatment could not aid recovery of BRB damage and microglial activation (identified by increased CD11b^+^ CD80^+^ cells induced by overexpressing VEGF in the mouse retina) (Arima et al., 2020). Additionally, the expression of rho kinase (ROCK), a protein that contributes to modulating microglial inflammatory responses, was increased in these retinas (Roser et al., 2017; Arima et al., 2020). Interestingly, the application of a ROCK inhibitor was able to recover the aforementioned changes induced by increased levels of VEGF including reduced CD11b^+^ CD80^+^ cells (microglial activation) (Arima et al., 2020).

## Conclusion

Microglia, the resident macrophages of the CNS, plays a crucial role in innate immunity and neuroinflammatory pathologies. Microglia act as a double-edged sword exerting both neuroprotective and neurotoxic functions in response to age- and retinal degeneration-associated stimuli in the brain and retina. Senescent alterations of microglia have been linked to retinal neuroinflammation that has been increasingly recognised as a key contributor to initiate age-dependent retinal neurodegeneration.

Generally, with ageing, morphological changes were indicative of increased activated or amoeboid microglia, further confirmed by increases in expression of markers of microglial activation, such as IB4 or CD68. Retinal microglia are also reduced in their motility with age. Interestingly, in models and cases of glaucoma, AMD and DR, a similar type of microglial activation with their morphology and expression of activation markers, was commonly observed. These investigations have shown that such microglial responses also resulted in, for example: RGC loss in glaucoma; photoreceptor loss, drusen formation and CNV in AMD; and leaky vessels and increased levels of VEGF in DR. This could have major applications in the use of less invasive techniques to identify and monitor disease activity via the eye. For instance, the vitreous humour could be used for cytokine profiling, since microglial activation in ageing and disease can manipulate levels of cytokines. Additionally, with advances of retinal imaging, retinal microglial morphologies could be monitored to track age- or disease-associated microglial activation. This could also be a major advancement in therapeutic research of retinal diseases, enabling de-risking and acceleration of clinical development programmes by using the eye to monitor intervention efficacies.

Despite this, there are also findings of contradicting evidence. Although these contradictions could have been due to factors such as different disease models used, this emphasizes the need to establish and utilize more standardized methods to profile microglial activation in the retina. As of now, it is still difficult to delineate between microglial activation profiles in these retinal diseases compared to normal ageing. This reflects the need for future investigations to look at both ageing in disease and the onset of disease at different ages, in order to reveal the independent and interlinked mechanisms of age-associated and disease-associated microglial changes. Overall, this may increase the likelihood of identifying reliable and accurate microglia-associated biomarkers to identify and track disease activity.

## Author Contributions

LG: manuscript planning, writing, and editing. SC: manuscript writing and editing. PB: figure and table preparations and editing. MC: conceptualisation, supervision, and editing. All authors have read and approved the final manuscript.

## Conflict of Interest

The authors declare that the research was conducted in the absence of any commercial or financial relationships that could be construed as a potential conflict of interest.

## Publisher’s Note

All claims expressed in this article are solely those of the authors and do not necessarily represent those of their affiliated organizations, or those of the publisher, the editors and the reviewers. Any product that may be evaluated in this article, or claim that may be made by its manufacturer, is not guaranteed or endorsed by the publisher.

## References

[B1] Alliot F. Godin I. Pessac B. (1999). Microglia derive from progenitors, originating from the yolk sac, and which proliferate in the brain. *Dev. Brain Res.* 117 145–152. 10.1016/S0165-3806(99)00113-3 10567732

[B2] Allison K. Patel D. Alabi O. (2020). Epidemiology of Glaucoma: the Past, Present, and Predictions for the Future. *Cureus* 12:e11686. 10.7759/cureus.11686 33391921 PMC7769798

[B3] Anderson S. R. Roberts J. M. Zhang J. Steele M. R. Romero C. O. Bosco A. (2019). Developmental Apoptosis Promotes a Disease-Related Gene Signature and Independence from CSF1R Signaling in Retinal Microglia. *Cell Rep.* 27 2002–2013.e5. 10.1016/J.CELREP.2019.04.062 31091440 PMC6544177

[B4] Arima M. Nakao S. Yamaguchi M. Feng H. Fujii Y. Shibata K. (2020). Claudin-5 Redistribution Induced by Inflammation Leads to Anti-VEGF–Resistant Diabetic Macular Edema. *Diabetes* 69 981–999. 10.2337/DB19-1121 32139595

[B5] Askew K. Li K. Olmos-Alonso A. Garcia-Moreno F. Liang Y. Richardson P. (2017). Coupled Proliferation and Apoptosis Maintain the Rapid Turnover of Microglia in the Adult Brain. *Cell Rep.* 18 391–405. 10.1016/j.celrep.2016.12.041 28076784 PMC5263237

[B6] Bialas A. Stevens B. (2013). TGF-β signaling regulates neuronal C1q expression and developmental synaptic refinement. *Nat. Neurosci.* 16 1773–1782. 10.1038/NN.3560 24162655 PMC3973738

[B7] Bliederhaeuser C. Grozdanov V. Speidel A. Zondler L. Ruf W. P. Bayer H. (2016). Age-dependent defects of alpha-synuclein oligomer uptake in microglia and monocytes. *Acta Neuropathol.* 131 379–391. 10.1007/s00401-015-1504-2 26576561

[B8] Boche D. Perry V. H. Nicoll J. A. R. (2013). Review: activation patterns of microglia and their identification in the human brain. *Neuropathol. Appl. Neurobiol.* 39 3–18. 10.1111/nan.12011 23252647

[B9] Borucki D. M. Toutonji A. Couch C. Mallah K. Rohrer B. Tomlinson S. (2020). Complement-Mediated Microglial Phagocytosis and Pathological Changes in the Development and Degeneration of the Visual System. *Front. Immunol.* 11:566892. 10.3389/fimmu.2020.566892 33072106 PMC7541817

[B10] Bosco A. Anderson S. R. Roberts J. M. Romero C. O. Steele M. R. Vetter M. L. (2019). Retinal microglia acquire a disease-associated transcriptome in chronic mouse glaucoma, which intensifies with neuroprotective complement inhibition. *Invest. Ophthalmol. Vis. Sci.* 60 4002–4002.31560766

[B11] Caldeira C. Cunha C. Vaz A. R. Falcão A. S. Barateiro A. Seixas E. (2017). Key aging-associated alterations in primary microglia response to beta-amyloid stimulation. *Front. Aging Neurosci.* 9:277. 10.3389/fnagi.2017.00277 28912710 PMC5583148

[B12] Charles A. Janeway J. Travers P. Walport M. Shlomchik M. J. (2001). “*The major histocompatibility complex and its functions,” in *Immunobiology: The Immune System in Health and Disease*, 5th Edn, eds Austin P. Lawrence E. (New York: Garland Science).

[B13] Chen M. Muckersie E. Forrester J. V. Xu H. (2010). Immune Activation in Retinal Aging: a Gene Expression Study. *Investig. Opthalmol. Vis. Sci.* 51:5888. 10.1167/iovs.09-5103 20538981

[B14] Chen M. Xu H. (2015). Parainflammation, chronic inflammation, and age-related macular degeneration. *J. Leukoc. Biol.* 98 713–725. 10.1189/jlb.3RI0615-239R 26292978 PMC4733662

[B15] Chen X. Zhou H. Gong Y. Wei S. Zhang M. (2015). Early spatiotemporal characterization of microglial activation in the retinas of rats with streptozotocin-induced diabetes. *Graefes Arch. Clin. Exp. Ophthalmol.* 253 519–525. 10.1007/s00417-014-2727-y 25023148

[B16] Cheng S. Wang H.-N. Xu L.-J. Li F. Miao Y. Lei B. (2021). Soluble tumor necrosis factor-alpha-induced hyperexcitability contributes to retinal ganglion cell apoptosis by enhancing Nav1.6 in experimental glaucoma. *J. Neuroinflammation* 18:182. 10.1186/S12974-021-02236-6 34419081 PMC8380326

[B17] Chidlow G. Ebneter A. Wood J. P. M. Casson R. J. (2016). Evidence supporting an association between expression of major histocompatibility complex II by microglia and optic nerve degeneration during experimental glaucoma. *J. Glaucoma* 25 681–691. 10.1097/IJG.0000000000000447 27253969

[B18] Chidlow G. Wood J. P. M. Manavis J. Finnie J. Casson R. J. (2017). Investigations into Retinal Pathology in the Early Stages of a Mouse Model of Alzheimer’s Disease. *J. Alzheimers Dis.* 56 655–675. 10.3233/JAD-160823 28035930 PMC5271427

[B19] Chirco K. R. Sohn E. H. Stone E. M. Tucker B. A. Mullins R. F. (2016). Structural and molecular changes in the aging choroid: implications for age-related macular degeneration. *Eye* 311 10–25. 10.1038/eye.2016.216 27716746 PMC5233940

[B20] Cho M. H. Cho K. Kang H. J. Jeon E. Y. Kim H. S. Kwon H. J. (2014). Autophagy in microglia degrades extracellular β-amyloid fibrils and regulates the NLRP3 inflammasome. *Autophagy* 10 1761–1775. 10.4161/auto.29647 25126727 PMC4198361

[B21] Combadière C. Feumi C. Raoul W. Keller N. Rodéro M. Pézard A. (2007). CX3CR1-dependent subretinal microglia cell accumulation is associated with cardinal features of age-related macular degeneration. *J. Clin. Invest.* 117 2920–2928. 10.1172/JCI31692 17909628 PMC1994614

[B22] Conde J. R. Streit W. J. (2006). Effect of aging on the microglial response to peripheral nerve injury. *Neurobiol. Aging* 27 1451–1461. 10.1016/j.neurobiolaging.2005.07.012 16159684

[B23] Damani M. R. Zhao L. Fontainhas A. M. Amaral J. Fariss R. N. Wong W. T. (2011). Age-related alterations in the dynamic behavior of microglia. *Aging Cell* 10 263–276. 10.1111/j.1474-9726.2010.00660.x 21108733 PMC3056927

[B24] Daria A. Colombo A. Llovera G. Hampel H. Willem M. Liesz A. (2017). Young microglia restore amyloid plaque clearance of aged microglia. *EMBO J.* 36 583–603. 10.15252/embj.201694591 28007893 PMC5331757

[B25] Davies D. S. Ma J. Jegathees T. Goldsbury C. (2017). Microglia show altered morphology and reduced arborization in human brain during aging and Alzheimer’s disease. *Brain Pathol.* 27 795–808. 10.1111/bpa.12456 27862631 PMC8029278

[B26] Davis B. M. Crawley L. Pahlitzsch M. Javaid F. Cordeiro M. F. (2016). Glaucoma: the retina and beyond. *Acta Neuropathol.* 132 807–826. 10.1007/s00401-016-1609-2 27544758 PMC5106492

[B27] Davis B. M. Salinas-Navarro M. Cordeiro M. F. Moons L. De Groef L. (2017). Characterizing microglia activation: a spatial statistics approach to maximize information extraction. *Sci. Rep.* 7:1576. 10.1038/s41598-017-01747-8 28484229 PMC5431479

[B28] de Hoz R. Gallego B. I. Ramírez A. I. Rojas B. Salazar J. J. Valiente-Soriano F. J. (2013). Rod-Like Microglia Are Restricted to Eyes with Laser-Induced Ocular Hypertension but Absent from the Microglial Changes in the Contralateral Untreated Eye. *PLoS One* 8:e83733. 10.1371/journal.pone.0083733 24367610 PMC3867486

[B29] de Hoz R. Ramírez A. I. González-Martín R. Ajoy D. Rojas B. Salobrar-Garcia E. (2018). Bilateral early activation of retinal microglial cells in a mouse model of unilateral laser-induced experimental ocular hypertension. *Exp. Eye Res.* 171 12–29. 10.1016/j.exer.2018.03.006 29526796

[B30] Deczkowska A. Keren-Shaul H. Weiner A. Colonna M. Schwartz M. Amit I. (2018). Disease-Associated Microglia: a Universal Immune Sensor of Neurodegeneration. *Cell* 173 1073–1081. 10.1016/j.cell.2018.05.003 29775591

[B31] Deng Y. Y. Lu J. Ling E. A. Kaur C. (2009). Monocyte chemoattractant protein-1 (MCP-1) produced via NF-κB signaling pathway mediates migration of amoeboid microglia in the periventricular white matter in hypoxic neonatal rats. *Glia* 57 604–621. 10.1002/glia.20790 18942743

[B32] Dietze J. Blair K. Havens S. J. (2022). *Glaucoma.* Available online at: https://www.ncbi.nlm.nih.gov/books/NBK538217/ (Accessed February 2, 2022).

[B33] Dumitrascu O. M. Lyden P. D. Torbati T. Sheyn J. Sherzai A. Sherzai D. (2020). Sectoral segmentation of retinal amyloid imaging in subjects with cognitive decline. *Alzheimers Dement.* 12:e12109. 10.1002/dad2.12109 33015311 PMC7521595

[B34] Elkabes S. DiCicco-Bloom E. M. Black I. B. (1996). Brain microglia/macrophages express neurotrophins that selectively regulate microglial proliferation and function. *J. Neurosci.* 16 2508–2521. 10.1523/jneurosci.16-08-02508.1996 8786427 PMC6578768

[B35] Fernández-Albarral J. A. Salobrar-Garcia E. López-Cuenca I. Rojas Lozano M. P. José Salazar Corral J. De Hoz R. (2021a). Microglial changes in healthy mice retina in an early aging stage. *Acta Ophthalmol.* 99:S265. 10.1111/j.1755-3768.2020.0138

[B36] Fernández-Albarral J. A. Salazar J. J. de Hoz R. Marco E. M. Martín-Sánchez B. Flores-Salguero E. (2021b). Retinal Molecular Changes Are Associated with Neuroinflammation and Loss of RGCs in an Experimental Model of Glaucoma. *Int. J. Mol. Sci.* 22:2066. 10.3390/IJMS22042066 33669765 PMC7922243

[B37] Floden A. M. Combs C. K. (2011). Microglia Demonstrate Age-Dependent Interaction with Amyloid-β Fibrils. *J. Alzheimers Dis.* 25 279–293. 10.3233/JAD-2011-101014 21403390 PMC3295838

[B38] Franco-Bocanegra D. K. McAuley C. Nicoll J. A. R. Boche D. (2019). Molecular Mechanisms of Microglial Motility: changes in Ageing and Alzheimer’s Disease. *Cells* 8:639. 10.3390/cells8060639 31242692 PMC6627151

[B39] Gabandé-Rodríguez E. Keane L. Capasso M. (2020). Microglial phagocytosis in aging and Alzheimer’s disease. *J. Neurosci. Res.* 98 284–298. 10.1002/jnr.24419 30942936

[B40] Gehrig A. Langmann T. Horling F. Janssen A. Bonin M. Walter M. (2007). Genome-wide expression profiling of the retinoschisin-deficient retina in early postnatal mouse development. *Invest. Ophthalmol. Vis. Sci.* 48 891–900. 10.1167/IOVS.06-0641 17251492

[B41] Graham M. L. Janecek J. L. Kittredge J. A. Hering B. J. Schuurman H.-J. (2011). The streptozotocin-induced diabetic nude mouse model: differences between animals from different sources. *Comp. Med.* 61 356–360. 22330251 PMC3155402

[B42] Gupta N. Brown K. E. Milam A. H. (2003). Activated microglia in human retinitis pigmentosa, late-onset retinal degeneration, and age-related macular degeneration. *Exp. Eye Res.* 76 463–471. 10.1016/S0014-4835(02)00332-9 12634111

[B43] Guzman-Martinez L. Maccioni R. B. Andrade V. Navarrete L. P. Pastor M. G. Ramos-Escobar N. (2019). Neuroinflammation as a Common Feature of Neurodegenerative Disorders. *Front. Pharmacol.* 10:1008. 10.3389/fphar.2019.01008 31572186 PMC6751310

[B44] Hachana S. Pouliot M. Couture R. Vaucher E. (2020). Diabetes-Induced Inflammation and Vascular Alterations in the Goto–Kakizaki Rat Retina. *Curr. Eye Res.* 45 965–974. 10.1080/02713683.2020.1712730 31902231

[B45] Han J. Wang M. Ren M. Lou H. (2017). Contributions of triggering-receptor-expressed-on-myeloid-cells-2 to neurological diseases. *Int. J. Neurosci.* 127 368–375. 10.1080/00207454.2016.1264072 27871212

[B46] Hanisch U.-K. (2002). Microglia as a source and target of cytokines. *Glia* 40 140–155. 10.1002/glia.10161 12379902

[B47] Hanisch U.-K. Kettenmann H. (2007). Microglia: active sensor and versatile effector cells in the normal and pathologic brain. *Nat. Neurosci.* 10 1387–1394. 10.1038/nn1997 17965659

[B48] Hanke M. L. Kielian T. (2011). Toll-like receptors in health and disease in the brain: mechanisms and therapeutic potential. *Clin. Sci.* 121 367–387. 10.1042/CS20110164 21745188 PMC4231819

[B49] Harada C. Harada T. Quah H. M. A. Maekawa F. Yoshida K. Ohno S. (2003). Potential role of glial cell line-derived neurotrophic factor receptors in Müller glial cells during light-induced retinal degeneration. *Neuroscience* 122 229–235. 10.1016/S0306-4522(03)00599-2 14596863

[B50] Hefendehl J. K. Neher J. J. Sühs R. B. Kohsaka S. Skodras A. Jucker M. (2014). Homeostatic and injury-induced microglia behavior in the aging brain. *Aging Cell* 13 60–69. 10.1111/acel.12149 23953759 PMC4326865

[B51] Hendrickx D. A. E. Schuurman K. G. van Draanen M. Hamann J. Huitinga I. (2014). Enhanced uptake of multiple sclerosis-derived myelin by THP-1 macrophages and primary human microglia. *J. Neuroinflammation* 11:64. 10.1186/1742-2094-11-64 24684721 PMC4108133

[B52] Hide I. Tanaka M. Inoue A. Nakajima K. Kohsaka S. Inoue K. (2000). Extracellular ATP triggers tumor necrosis factor-α release from rat microglia. *J. Neurochem.* 75 965–972. 10.1046/j.1471-4159.2000.0750965.x 10936177

[B53] Holloway O. G. Canty A. J. King A. E. Ziebell J. M. (2019). Rod microglia and their role in neurological diseases. *Semin. Cell Dev. Biol.* 94 96–103. 10.1016/j.semcdb.2019.02.005 30826549

[B54] Honda S. Sasaki Y. Ohsawa K. Imai Y. Nakamura Y. Inoue K. (2001). Extracellular ATP or ADP induce chemotaxis of cultured microglia through Gi/o-coupled P2Y receptors. *J. Neurosci.* 21 1975–1982. 10.1523/jneurosci.21-06-01975.2001 11245682 PMC6762617

[B55] Inafuku S. Klokman G. Connor K. M. (2018). The Alternative Complement System Mediates Cell Death in Retinal Ischemia Reperfusion Injury. *Front. Mol. Neurosci.* 11:278. 10.3389/fnmol.2018.00278 30174588 PMC6107794

[B56] Indaram M. Ma W. Zhao L. Fariss R. N. Rodriguez I. R. Wong W. T. (2015). 7-Ketocholesterol Increases Retinal Microglial Migration, Activation and Angiogenicity: a Potential Pathogenic Mechanism Underlying Age-related Macular Degeneration. *Sci. Rep.* 5:9144. 10.1038/srep09144 25775051 PMC4360733

[B57] Inoue K. (2002). Microglial activation by purines and pyrimidines. *Glia* 40 156–163. 10.1002/glia.10150 12379903

[B58] Karlstetter M. Scholz R. Rutar M. Wong W. T. Provis J. M. Langmann T. (2015). Retinal microglia: just bystander or target for therapy? *Prog. Retin. Eye Res.* 45 30–57. 10.1016/j.preteyeres.2014.11.004 25476242

[B59] Karperien A. Ahammer H. Jelinek H. F. (2013). Quantitating the subtleties of microglial morphology with fractal analysis. *Front. Cell. Neurosci.* 7:3. 10.3389/fncel.2013.00003 23386810 PMC3558688

[B60] Keren-Shaul H. Spinrad A. Weiner A. Matcovitch-Natan O. Dvir-Szternfeld R. Ulland T. K. (2017). A Unique Microglia Type Associated with Restricting Development of Alzheimer’s Disease. *Cell* 169 1276–1290.e17. 10.1016/j.cell.2017.05.018 28602351

[B61] Khurana B. (2002). Functions of LIM proteins in cell polarity and chemotactic motility. *EMBO J.* 21 5331–5342. 10.1093/emboj/cdf550 12374734 PMC129082

[B62] Kinuthia U. M. Wolf A. Langmann T. (2020). Microglia and Inflammatory Responses in Diabetic Retinopathy. *Front. Immunol.* 11:2888. 10.3389/fimmu.2020.564077 33240260 PMC7681237

[B63] Kohno H. Chen Y. Kevany B. M. Pearlman E. Miyagi M. Maeda T. (2013). Photoreceptor Proteins Initiate Microglial Activation via Toll-like Receptor 4 in Retinal Degeneration Mediated by All-trans-retinal. *J. Biol. Chem.* 288 15326–15341. 10.1074/jbc.M112.448712 23572532 PMC3663552

[B64] Koronyo Y. Biggs D. Barron E. Boyer D. S. Pearlman J. A. Au W. J. (2017). Retinal amyloid pathology and proof-of-concept imaging trial in Alzheimer’s disease. *JCI Insight* 2:e93621. 10.1172/jci.insight.93621 28814675 PMC5621887

[B65] Krasemann S. Madore C. Cialic R. Baufeld C. Calcagno N. El Fatimy R. (2017). The TREM2-APOE Pathway Drives the Transcriptional Phenotype of Dysfunctional Microglia in Neurodegenerative Diseases. *Immunity* 47 566–581.e9. 10.1016/j.immuni.2017.08.008 28930663 PMC5719893

[B66] Langmann T. (2007). Microglia activation in retinal degeneration. *J. Leukoc. Biol.* 81 1345–1351. 10.1189/jlb.0207114 17405851

[B67] Lee J. E. Liang K. J. Fariss R. N. Wong W. T. (2008). Ex Vivo Dynamic Imaging of Retinal Microglia Using Time-Lapse Confocal Microscopy. *Invest. Ophthalmol. Vis. Sci.* 49 4169–4176. 10.1167/iovs.08-2076 18487378 PMC2652634

[B68] Lehnardt S. Massillon L. Follett P. Jensen F. E. Ratan R. Rosenberg P. A. (2003). Activation of innate immunity in the CNS triggers neurodegeneration through a Toll-like receptor 4-dependent pathway. *Proc. Natl. Acad. Sci. U. S. A.* 100 8514–8519. 10.1073/PNAS.1432609100 12824464 PMC166260

[B69] Li J. Welchowski T. Schmid M. Mauschitz M. M. Holz F. G. Finger R. P. (2020). Prevalence and incidence of age-related macular degeneration in Europe: a systematic review and meta-analysis. *Br. J. Ophthalmol.* 104 1077–1084. 10.1136/BJOPHTHALMOL-2019-314422 31712255

[B70] Li W. (2013). Phagocyte dysfunction, tissue aging and degeneration. *Ageing Res. Rev.* 12 1005–1012. 10.1016/j.arr.2013.05.006 23748186 PMC3842398

[B71] Li X. Yu Z.-W. Li H.-Y. Yuan Y. Gao X.-Y. Kuang H.-Y. (2021). Retinal microglia polarization in diabetic retinopathy. *Vis. Neurosci.* 38:E006. 10.1017/S0952523821000031 33934736

[B72] Liang K. J. Lee J. E. Wang Y. D. Ma W. Fontainhas A. M. Fariss R. N. (2009). Regulation of Dynamic Behavior of Retinal Microglia by CX3CR1 Signaling. *Investig. Opthalmol. Vis. Sci.* 50:4444. 10.1167/iovs.08-3357 19443728 PMC2749316

[B73] Liao D. Grossi F. V. El Mehdi D Gerber M. R. Brown D. M. Heier J. S. (2020). Complement C3 Inhibitor Pegcetacoplan for Geographic Atrophy Secondary to Age-Related Macular Degeneration: a Randomized Phase 2 Trial. *Ophthalmology* 127 186–195. 10.1016/J.OPHTHA.2019.07.011 31474439

[B74] Ma W. Silverman S. M. Zhao L. Villasmil R. Campos M. M. Amaral J. (2019). Absence of TGFβ signaling in retinal microglia induces retinal degeneration and exacerbates choroidal neovascularization. *Elife* 8:e42049. 10.7554/eLife.42049 30666961 PMC6342522

[B75] Ma W. Wong W. T. (2016). Aging changes in retinal microglia and their relevance to age-related retinal disease. *Adv. Exp. Med. Biol.* 854 73–78. 10.1007/978-3-319-17121-0_11 26427396 PMC4696750

[B76] Madeira M. H. Boia R. Santos P. F. Ambrósio A. F. Santiago A. R. (2015). Contribution of Microglia-Mediated Neuroinflammation to Retinal Degenerative Diseases. *Mediators Inflamm.* 2015:673090. 10.1155/2015/673090 25873768 PMC4385698

[B77] Mantovani A. Sica A. Sozzani S. Allavena P. Vecchi A. Locati M. (2004). The chemokine system in diverse forms of macrophage activation and polarization. *Trends Immunol.* 25 677–686. 10.1016/j.it.2004.09.015 15530839

[B78] Marín-Teva J. L. Almendros A. Calvente R. Cuadros M. A. Navascués J. (1998). Tangential migration of ameboid microglia in the developing quail retina: mechanism of migration and migratory behavior. *Glia* 22 31–52. 10.1002/(sici)1098-1136(199801)22:1<31::aid-glia4<3.0.co;2-b 9436786

[B79] Marín-Teva J. L. Almendros A. Calvente R. Cuadros M. A. Navascués J. (1999). Proliferation of actively migrating ameboid microglia in the developing quail retina. *Anat. Embryol.* 200 289–300. 10.1007/s004290050280 10463344

[B80] Marsh B. J. Williams-Karnesky R. L. Stenzel-Poore M. P. (2009). Toll-like receptor signaling in endogenous neuroprotection and stroke. *Neuroscience* 158 1007–1020. 10.1016/j.neuroscience.2008.07.067 18809468 PMC2674023

[B81] Mazaheri F. Snaidero N. Kleinberger G. Madore C. Daria A. Werner G. (2017). TREM 2 deficiency impairs chemotaxis and microglial responses to neuronal injury. *EMBO Rep.* 18 1186–1198. 10.15252/embr.201743922 28483841 PMC5494532

[B82] McMonnies C. W. (2017). Glaucoma history and risk factors. *J. Optom.* 10 71–78. 10.1016/j.optom.2016.02.003 27025415 PMC5383456

[B83] Miller J. W. D’Anieri L. L. Husain D. Miller J. B. Vavvas D. G. (2021). Age-Related Macular Degeneration (AMD): a View to the Future. *J. Clin. Med.* 10:1124. 10.3390/JCM10051124 33800285 PMC7962647

[B84] Nakajima K. Tohyama Y. Kohsaka S. Kurihara T. (2001). Ability of rat microglia to uptake extracellular glutamate. *Neurosci. Lett.* 307 171–174. 10.1016/S0304-3940(01)01943-7 11438391

[B85] Namekata K. Guo X. Kimura A. Arai N. Harada C. Harada T. (2019). DOCK8 is expressed in microglia, and it regulates microglial activity during neurodegeneration in murine disease models. *J. Biol. Chem.* 294 13421–13433. 10.1074/jbc.RA119.007645 31337702 PMC6737224

[B86] Ngolab J. Donohue M. Belsha A. Salazar J. Cohen P. Jaiswal S. (2021). Feasibility study for detection of retinal amyloid in clinical trials: the Anti-Amyloid Treatment in Asymptomatic Alzheimer’s Disease (A4) trial. *Alzheimers Dement.* 13:e12199. 10.1002/dad2.12199 34430703 PMC8369843

[B87] Nimmerjahn A. Kirchhoff F. Helmchen F. (2005). Resting Microglial Cells Are Highly Dynamic Surveillants of Brain Parenchyma in Vivo. *Science* 308 1314–1318. 10.1126/science.1110647 15831717

[B88] Njie E. G. Boelen E. Stassen F. R. Steinbusch H. W. Borchelt D. R. Streit W. J. (2012). Ex vivo cultures of microglia from young and aged rodent brain reveal age-related changes in microglial function. *Neurobiol. Aging* 33 195.e1–12. 10.1016/j.neurobiolaging.2010.05.008 20580465 PMC4162517

[B89] Noailles A. Fernández-Sánchez L. Lax P. Cuenca N. (2014). Microglia activation in a model of retinal degeneration and TUDCA neuroprotective effects. *J. Neuroinflammation* 11:186. 10.1186/s12974-014-0186-3 25359524 PMC4221719

[B90] Omri S. Behar-Cohen F. de Kozak Y. Sennlaub F. Verissimo L. M. Jonet L. (2011). Microglia/Macrophages Migrate through Retinal Epithelium Barrier by a Transcellular Route in Diabetic Retinopathy: role of PKCζ in the Goto Kakizaki Rat Model. *Am. J. Pathol.* 179:942. 10.1016/J.AJPATH.2011.04.018 21712024 PMC3157258

[B91] Orihuela R. McPherson C. A. Harry G. J. (2016). Microglial M1/M2 polarization and metabolic states. *Br. J. Pharmacol.* 173 649–665. 10.1111/bph.13139 25800044 PMC4742299

[B92] Paolicelli R. C. Bolasco G. Pagani F. Maggi L. Scianni M. Panzanelli P. (2011). Synaptic pruning by microglia is necessary for normal brain development. *Science* 333 1456–1458. 10.1126/science.1202529 21778362

[B93] Poliani P. L. Wang Y. Fontana E. Robinette M. L. Yamanishi Y. Gilfillan S. (2015). TREM2 sustains microglial expansion during aging and response to demyelination. *J. Clin. Invest.* 125 2161–2170. 10.1172/JCI77983 25893602 PMC4463196

[B94] Raivich G. Jones L. L. Werner A. Blüthmann H. Doetschmann T. Kreutzberg G. W. (1999). Molecular signals for glial activation: pro- and anti-inflammatory cytokines in the injured brain. *Acta Neurochir. Suppl.* 73 21–30. 10.1007/978-3-7091-6391-7_4 10494337

[B95] Ramírez A. I. de Hoz R. Fernández-Albarral J. A. Salobrar-Garcia E. Rojas B. Valiente-Soriano F. J. (2020). Time course of bilateral microglial activation in a mouse model of laser-induced glaucoma. *Sci. Rep.* 10:4890. 10.1038/s41598-020-61848-9 32184450 PMC7078298

[B96] Ramirez A. I. de Hoz R. Salobrar-Garcia E. Salazar J. J. Rojas B. Ajoy D. (2017). The Role of Microglia in Retinal Neurodegeneration: Alzheimer’s Disease, Parkinson, and Glaucoma. *Front. Aging Neurosci.* 9:214. 10.3389/fnagi.2017.00214 28729832 PMC5498525

[B97] Rangasamy S. McGuire P. G. Franco Nitta C. Monickaraj F. Oruganti S. R. Das A. (2014). Chemokine Mediated Monocyte Trafficking into the Retina: role of Inflammation in Alteration of the Blood-Retinal Barrier in Diabetic Retinopathy. *PLoS One* 9:e108508. 10.1371/journal.pone.0108508 25329075 PMC4203688

[B98] Rashid K. Akhtar-Schaefer I. Langmann T. (2019). Microglia in Retinal Degeneration. *Front. Immunol.* 10:1975. 10.3389/fimmu.2019.01975 31481963 PMC6710350

[B99] Ritzel R. M. Doran S. J. Glaser E. P. Meadows V. E. Faden A. I. Stoica B. A. (2019). Old age increases microglial senescence, exacerbates secondary neuroinflammation, and worsens neurological outcomes after acute traumatic brain injury in mice. *Neurobiol. Aging* 77 194–206. 10.1016/j.neurobiolaging.2019.02.010 30904769 PMC6486858

[B100] Rodriguez I. R. Clark M. E. Lee J. W. Curcio C. A. (2014). 7-ketocholesterol accumulates in ocular tissues as a consequence of aging and is present in high levels in drusen. *Exp. Eye Res.* 128 151–155. 10.1016/J.EXER.2014.09.009 25261634 PMC4254360

[B101] Roser A.-E. Tönges L. Lingor P. (2017). Modulation of Microglial Activity by Rho-Kinase (ROCK) Inhibition as Therapeutic Strategy in Parkinson’s Disease and Amyotrophic Lateral Sclerosis. *Front. Aging Neurosci.* 9:94. 10.3389/fnagi.2017.00094 28420986 PMC5378706

[B102] Saab A. S. Nave K.-A. (2017). Myelin dynamics: protecting and shaping neuronal functions. *Curr. Opin. Neurobiol.* 47 104–112. 10.1016/j.conb.2017.09.013 29065345

[B103] Safaiyan S. Kannaiyan N. Snaidero N. Brioschi S. Biber K. Yona S. (2016). Age-related myelin degradation burdens the clearance function of microglia during aging. *Nat. Neurosci.* 19 995–998. 10.1038/nn.4325 27294511 PMC7116794

[B104] Santos A. M. Calvente R. Tassi M. Carrasco M. C. Martín-Oliva D. Marín-Teva J. L. (2008). Embryonic and postnatal development of microglial cells in the mouse retina. *J. Comp. Neurol.* 506 224–239. 10.1002/cne.21538 18022954

[B105] Sharma L. Wu W. Dholakiya S. L. Gorasiya S. Wu J. Sitapara R. (2014). Assessment of phagocytic activity of cultured macrophages using fluorescence microscopy and flow cytometry. *Methods Mol. Biol.* 1172 137–145. 10.1007/978-1-4939-0928-5_12 24908301

[B106] Sheu S. Sakamoto T. Osusky R. Wang H. M. Ogden T. E. Ryan S. J. (1994). Transforming growth factor-beta regulates human retinal pigment epithelial cell phagocytosis by influencing a protein kinase C-dependent pathway. *Graefes Arch. Clin. Exp. Ophthalmol.* 232 695–701. 10.1007/BF00171387 7531168

[B107] Shobin E. Bowley M. P. Estrada L. I. Heyworth N. C. Orczykowski M. E. Eldridge S. A. (2017). Microglia activation and phagocytosis: relationship with aging and cognitive impairment in the rhesus monkey. *Geroscience* 39 199–220. 10.1007/s11357-017-9965-y 28238188 PMC5411373

[B108] Sierra A. Abiega O. Shahraz A. Neumann H. (2013). Janus-faced microglia: beneficial and detrimental consequences of microglial phagocytosis. *Front. Cell. Neurosci.* 7:6. 10.3389/fncel.2013.00006 23386811 PMC3558702

[B109] Silverman S. M. Wong W. T. (2018). Microglia in the Retina: roles in Development, Maturity, and Disease. *Annu. Rev. Vis. Sci.* 4 45–77. 10.1146/annurev-vision-091517-034425 29852094

[B110] Stefanova N. Fellner L. Reindl M. Masliah E. Poewe W. Wenning G. K. (2011). Toll-like receptor 4 promotes α-synuclein clearance and survival of nigral dopaminergic neurons. *Am. J. Pathol.* 179 954–963. 10.1016/j.ajpath.2011.04.013 21801874 PMC3157205

[B111] Streit W. J. (2002). Microglia as neuroprotective, immunocompetent cells of the CNS. *Glia* 40 133–139. 10.1002/glia.10154 12379901

[B112] Takahashi K. Rochford C. D. P. Neumann H. (2005). Clearance of apoptotic neurons without inflammation by microglial triggering receptor expressed on myeloid cells-2. *J. Exp. Med.* 201 647–657. 10.1084/jem.20041611 15728241 PMC2213053

[B113] Tosi G. Orlandini M. Galvagni F. (2018). The Controversial Role of TGF-β in Neovascular Age-Related Macular Degeneration Pathogenesis. *Int. J. Mol. Sci.* 19:3363. 10.3390/ijms19113363 30373226 PMC6275040

[B114] Vallat-Decouvelaere A. V. Chrétien F. Gras G. Le Pavec G. Dormont D. Gray F. (2003). Expression of Excitatory Amino Acid Transporter-1 in Brain Macrophages and Microglia of HIV-Infected Patients. A Neuroprotective Role for Activated Microglia? *J. Neuropathol. Exp. Neurol.* 62 475–485. 10.1093/jnen/62.5.475 12769187

[B115] Van Rossum D. Hanisch U. K. (2004). Microglia. *Metab. Brain Dis.* 19 393–411. 10.1023/B:MEBR.0000043984.73063.d8 15554430

[B116] Venezia S. Refolo V. Polissidis A. Stefanis L. Wenning G. K. Stefanova N. (2017). Toll-like receptor 4 stimulation with monophosphoryl lipid A ameliorates motor deficits and nigral neurodegeneration triggered by extraneuronal α-synucleinopathy. *Mol. Neurodegener.* 12:52. 10.1186/s13024-017-0195-7 28676095 PMC5496237

[B117] Vrabec F. (1975). Activated human retinal microglia under pathological conditions. *Albrecht Von Graefes Arch. Clin. Exp. Ophthalmol.* 196 49–60. 10.1007/BF00410026 1080638

[B118] Wallsh J. O. Gallemore R. P. (2021). Anti-VEGF-Resistant Retinal Diseases: a Review of the Latest Treatment Options. *Cells* 10:1049. 10.3390/cells10051049 33946803 PMC8145407

[B119] Wang K. Peng B. Lin B. (2014). Fractalkine receptor regulates microglial neurotoxicity in an experimental mouse glaucoma model. *Glia* 62 1943–1954. 10.1002/glia.22715 24989686

[B120] Wang M. Ma W. Zhao L. Fariss R. N. Wong W. T. (2011). Adaptive Müller cell responses to microglial activation mediate neuroprotection and coordinate inflammation in the retina. *J. Neuroinflammation* 8:173. 10.1186/1742-2094-8-173 22152278 PMC3251543

[B121] Wasserman J. K. Schlichter L. C. (2008). White matter injury in young and aged rats after intracerebral hemorrhage. *Exp. Neurol.* 214 266–275. 10.1016/j.expneurol.2008.08.010 18848934

[B122] Waugh N. Loveman E. Colquitt J. Royle P. Yeong J. L. Hoad G. (2018). Treatments for dry age-related macular degeneration and Stargardt disease: a systematic review. *Health Technol. Assess.* 22 1–168. 10.3310/hta22270 29846169 PMC5994642

[B123] Wolf S. A. Boddeke H. W. G. M. Kettenmann H. (2017). Microglia in Physiology and Disease. *Annu. Rev. Physiol.* 79 619–643. 10.1146/annurev-physiol-022516-034406 27959620

[B124] Wu J. Gao G. Shi F. Xie H. Yang Q. Liu D. (2021). Activated microglia–induced neuroinflammatory cytokines lead to photoreceptor apoptosis in Aβ-injected mice. *J. Mol. Med.* 99 713–728. 10.1007/s00109-021-02046-6 33575853

[B125] Wynne A. M. Henry C. J. Huang Y. Cleland A. Godbout J. P. (2010). Protracted downregulation of CX3CR1 on microglia of aged mice after lipopolysaccharide challenge. *Brain Behav. Immun.* 24 1190–1201. 10.1016/j.bbi.2010.05.011 20570721 PMC2939290

[B126] Xie H. Zhang C. Liu D. Yang Q. Tang L. Wang T. (2021). Erythropoietin protects the inner blood-retinal barrier by inhibiting microglia phagocytosis via Src/Akt/cofilin signalling in experimental diabetic retinopathy. *Diabetologia* 64 211–225. 10.1007/S00125-020-05299-X 33104828

[B127] Xu H. Chen M. (2017). Diabetic retinopathy and dysregulated innate immunity. *Vision Res.* 139 39–46. 10.1016/J.VISRES.2017.04.013 28571700

[B128] Xu H. Chen M. Manivannan A. Lois N. Forrester J. V. (2008). Age-dependent accumulation of lipofuscin in perivascular and subretinal microglia in experimental mice. *Aging Cell* 7 58–68. 10.1111/j.1474-9726.2007.00351.x 17988243

[B129] Yi W. Lu Y. Zhong S. Zhang M. Sun L. Dong H. (2020). A single-cell transcriptome atlas of the aging human and macaque retina. *Natl. Sci. Rev.* 8:nwaa179. 10.1093/nsr/nwaa179 34691611 PMC8288367

[B130] Yuan L. Neufeld A. H. (2001). Activated microglia in the human glaucomatous optic nerve head. *J. Neurosci. Res.* 64 523–532. 10.1002/jnr.1104 11391707

[B131] Yuan T. F. Liang Y. X. Peng B. Lin B. So K. F. (2015). Local proliferation is the main source of rod microglia after optic nerve transection. *Sci. Rep.* 51:10788. 10.1038/srep10788 26035780 PMC4649910

[B132] Yun J. H. (2021). Interleukin-1β induces pericyte apoptosis via the NF-κB pathway in diabetic retinopathy. *Biochem. Biophys. Res. Commun.* 546 46–53. 10.1016/J.BBRC.2021.01.108 33571904

[B133] Zeng H. (2008). Microglial Activation in Human Diabetic Retinopathy. *Arch. Ophthalmol.* 126:227. 10.1001/archophthalmol.2007.65 18268214

[B134] Zeng H. Y. Zhu X. A. Zhang C. Yang L. P. Wu L. M. Tso M. O. M. (2005). Identification of sequential events and factors associated with microglial activation, migration, and cytotoxicity in retinal degeneration in rd mice. *Invest. Ophthalmol. Vis. Sci.* 46 2992–2999. 10.1167/IOVS.05-0118 16043876

